# Importance of Incorporating Protein Flexibility in Molecule Modeling: A Theoretical Study on Type I^1/2^ NIK Inhibitors

**DOI:** 10.3389/fphar.2019.00345

**Published:** 2019-04-09

**Authors:** Chao Shen, Hui Liu, Xuwen Wang, Tailong Lei, Ercheng Wang, Lei Xu, Huidong Yu, Dan Li, Xiaojun Yao

**Affiliations:** ^1^College of Pharmaceutical Sciences, Zhejiang University, Hangzhou, China; ^2^School of Electrical and Information Engineering, Institute of Bioinformatics and Medical Engineering, Jiangsu University of Technology, Changzhou, China; ^3^Rongene Pharma Co., Ltd., Shenzhen, China; ^4^State Key Laboratory of Quality Research in Chinese Medicines, Macau University of Science and Technology, Macau, China

**Keywords:** NF-κB inducing kinase, NIK inhibitors, molecule modeling, binding mechanism, inhibitor design

## Abstract

NF-κB inducing kinase (NIK), which is considered as the central component of the non-canonical NF-κB pathway, has been proved to be an important target for the regulation of the immune system. In the past few years, NIK inhibitors with various scaffolds have been successively reported, among which type I^1/2^ inhibitors that can not only bind in the ATP-binding pocket at the DFG-in state but also extend into an additional back pocket, make up the largest proportion of the NIK inhibitors, and are worthy of more attention. In this study, an integration protocol that combines molecule docking, MD simulations, ensemble docking, MM/GB(PB)SA binding free energy calculations, and decomposition was employed to understand the binding mechanism of 21 tricyclic type I^1/2^ NIK inhibitors. It is found that the docking accuracy is largely dependent on the selection of docking protocols as well as the crystal structures. The predictions given by the ensemble docking based on multiple receptor conformations (MRCs) and the MM/GB(PB)SA calculations based on MD simulations showed higher linear correlations with the experimental data than those given by conventional rigid receptor docking (RRD) methods (Glide, GOLD, and Autodock Vina), highlighting the importance of incorporating protein flexibility in predicting protein–ligand interactions. Further analysis based on MM/GBSA demonstrates that the hydrophobic interactions play the most essential role in the ligand binding to NIK, and the polar interactions also make an important contribution to the NIK-ligand recognition. A deeper comparison of several pairs of representative derivatives reveals that the hydrophobic interactions are vitally important in the structural optimization of analogs as well. Besides, the H-bond interactions with some key residues and the large desolvation effect in the back pocket devote to the affinity distinction. It is expected that our study could provide valuable insights into the design of novel and potent type I^1/2^ NIK inhibitors.

## Introduction

Nuclear factor κB (NF-κB), which regulates the expression of a great number of genes that are critical for the regulation of apoptosis, tumorigenesis, inflammation, and a variety of autoimmune diseases, has gained considerable attention in the past two decades (Sun, 2017; Tegowski and Baldwin, 2018). Nowadays, it is known that the activation of NF-κB is mediated by two major signaling pathways, termed as the canonical and non-canonical NF-κB signaling pathways (Karin and Greten, 2005). The canonical pathway regulates the release and nuclear translocation of the canonical NF-κB subunits (such as NF-κB1 p50, RELA, and c-REL), whereas the non-canonical pathway is selectively responsible for the generation of NF-κB2 p52 (Bonizzi and Karin, 2004; Taniguchi and Karin, 2018).

The non-canonical signaling pathway is principally induced by a subset of tumor necrosis factor receptor (TNFR) family members [such as CD40, B cell activating factor receptor (BAFFR), and lymphotoxin-β receptor (LTβR)] (Vallabhapurapu et al., 2008; Vallabhapurapu and Karin, 2009). NF-κB inducing kinase (NIK), a serine/threonine kinase also known as MAP3K14, is considered as the central component of the non-canonical pathway (Malinin et al., 1997; Zarnegar et al., 2008). When activated, NIK can induce the activation of the downstream IκB kinase (IKKα), thereby leading to the processing of the inactive NF-κB2 p100 to its active p52 form (Senftleben et al., 2001; Xiao et al., 2001). Then, along with its main partner RELB, p52 can fully exert its biological functions including lymphoid organ development (Weih and Caamano, 2003), B cell maturation (Jellusova et al., 2013), lymphocyte recruitment (Muthuswamy et al., 2012), *etc*. Thus, NIK has become a potential therapeutic target and designing small-molecule inhibitors targeting NIK may provide a promising strategy for the intervention of the non-canonical signaling pathway as well as the regulation of the immune system (Cildir et al., 2016; Brightbill et al., 2018).

As far as we know, the NIK type I kinase inhibitors that just bind to the ATP-binding pocket at the active DFG(Asp-Phe-Gly)-in conformation and the type II inhibitors that occupy both the ATP-binding pocket and the adjacent hydrophobic pocket at an inactive DFG-out conformation represent two major classes of the kinase inhibitors (Mueller et al., 2015). Interestingly, most NIK inhibitors belong to type I^1/2^ inhibitors, an uncommon type that binds in the active state and extends into an additional back cavity as well. Since Li and co-workers (Li et al., 2013) reported the first type I^1/2^ NIK inhibitor derived from a type I hit identified by high-throughput screening and solved the first type I^1/2^ NIK-inhibitor co-crystal structure (PDB entry: 4IDV) in 2013, great progress has been achieved in the development of novel and potent type I^1/2^ NIK inhibitors (Castanedo et al., 2017; Kargbo, 2017; Blaquiere et al., 2018; Brightbill et al., 2018; Pippione et al., 2018). Until now, type I^1/2^ NIK inhibitors with various scaffolds, including pyrazole, azaindolylpyrimidine, pyrazolopyrimidine, thienopyrimidine, *etc*., have been published (De Leon-Boenig et al., 2012; Li et al., 2013; Castanedo et al., 2017; Blaquiere et al., 2018). As a common feature, their core regions can always form typical kinase hinge hydrogen bonding (H-bond) interactions with the protein backbone, such as Glu470^h^ (472^m^) and Leu472^h^ (474^m^), while the remaining part with an alkyne linker can pass the narrow channel embraced by gatekeeper Met469^h^ (471^m^) and catalytic Lys429^h^ (431^m^), and reach into the back pocket. The additional occupation of the back pocket makes a greater chance to form strong interactions with the surrounding residues, which may be favorable to design compounds with improved biological activity. However, up to now, no NIK inhibitor has been pushed into clinical trials, suggesting that discovery of novel NIK inhibitors is still urgently in need.

Due to the fact that experimental approaches are not fully available for all biological systems, computational methods such as molecule docking and molecular dynamics (MD) simulations have emerged as an essential alternative to elucidate the binding characteristics of bound ligands and guide the development of novel drugs (Alonso et al., 2006; Mortier et al., 2015; De Vivo et al., 2016). Hence, in this study, to better understand the binding mechanism of type I^1/2^ NIK inhibitors, based on a dataset with 29 tricyclic compounds extracted from the Castanedo's study (Castanedo et al., 2017), a theoretical case study involving several computational methods was conducted. Firstly, different docking tools were tried to predict the correct binding mode for each ligand. Nevertheless, just as various evidence proves, the binding of type I^1/2^ kinase inhibitors may be deeply influenced by the significant conformational plasticity of the receptor, and conventional rigid receptor docking (RRD) may not well reflect the real experimental activities (De Leon-Boenig et al., 2012; Kong et al., 2015, 2018; Pan et al., 2017). Therefore, several other methods that incorporate protein flexibility, such as induced-fit docking (IFD), MD simulations, and ensemble docking based on multiple receptor conformations (MRCs), were also adopted. Then, the Molecular Mechanics/Poisson-Boltzmann Surface Area (MM/PBSA) and Molecular Mechanics/Generalized Born Surface Area (MM/GBSA) approaches were employed to predict the binding affinities. Finally, integrating all the above computational technologies, a systematical analysis was carried out to further illustrate the binding characteristics of the selected molecules. Hopefully, our study could provide instructive guidance for the rational design of novel type I^1/2^ NIK inhibitors.

## Materials and Methods

### Preparation of Proteins and Ligands

**Figure 1:**
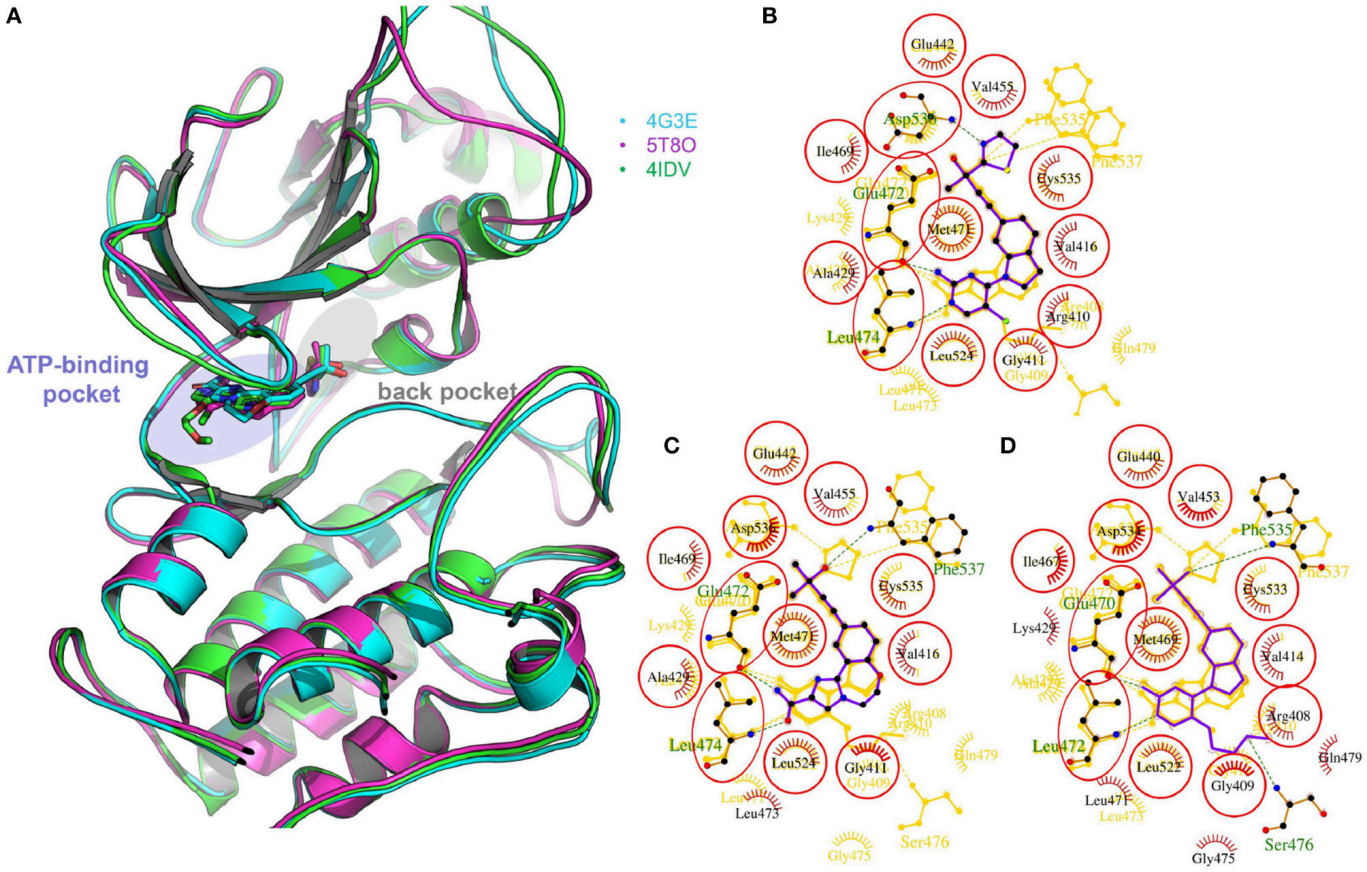
**(A)** The superimposition of two crystal structures of murine NIK domain (PDB entities: 4G3E and 5T8O) and one crystal structure of human NIK kinase domain (PDB entity: 4IDV). 4G3E, 5T8O, and 4IDV are colored in cyan, magenta and green, respectively. **(B–D)** The 2D protein-ligand interaction diagrams for 4G3E, 5T8O, and 4IDV, respectively. The ligand is colored in purple and the structures of the other two complexes are colored in gold. Red circles represent the structures aligned well in at least two complexes. The picture is produced by LigPlus (Laskowski and Swindells, 2011).

Two crystal structures of murine NIK kinase domain [PDB entries: 4G3E (De Leon-Boenig et al., 2012) and 5T8O (Castanedo et al., 2017)] and one crystal structure of human NIK kinase domain [PDB entity: 4IDV (Li et al., 2013)], whose bound ligands are type I^1/2^ kinase inhibitors, were put into use in this study. As murine NIK and human NIK have high sequence identify and all the residues within 5 Å to the bound ligands are the same (Figure 1), we supposed that the activities of the selected compounds should display a similar trend toward murine and human NIK. The missing loops were firstly added by the *Module/Refine Loop*s module in Chimera (Pettersen et al., 2004). Then, all these structures were processed by the *Protein Preparation Wizard* module (Sastry et al., 2013) in Schrödinger 2017, including removing waters and redundant chains, assigning bond orders, adding hydrogen atoms, filling in missing side chains, assigning H-bond networks, and finally minimizing the system by the OPLS3 force field until the root-mean-square-deviation (RMSD) of heavy atoms converged to 0.30 Å. The protonation states of residues were predicted by PROPKA (Olsson et al., 2011) at pH = 7.0. As for docking calculation, if the used program owns protein preparation function, its own function was utilized.

The information of the ligand dataset is summarized in Table 1. This series of compounds is composed of three similar tricyclic cores, including imidazobenzoxepin, thiabenzoxepin, and one bridged-bicyclic core. The structural modification is mainly made at the alkyne substitution and imidazole 5-substitution, which correspond to the hinge region binding and back pocket binding, respectively. Because the stereochemistry of some molecules was not determined, only 21 compounds were finally used in our study. The small molecules were sketched in Maestro and then prepared with the *Ligprep* (LigPrep, 2017) module in Schrödinger. The ionization states and tautomers were generated by *Epik* (Shelley et al., 2007; Greenwood et al., 2010) at pH = 7.0. The maximum number of the stereoisomers for each molecule was set to four and all the other parameters were set to default.

**Table 1:** Basic information of 29 tricyclic type I^1/2^ NIK Inhibitors.

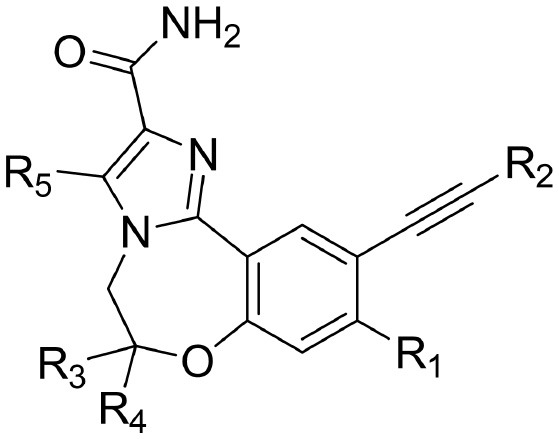						
**Compound**	**R1**	**R2**	**R3, R4**	**R5**	**NIK ADP-FP Ki (nM)**	**pKi**
**3**	H	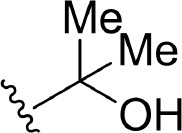	H, H	H	310 ± 110	6.509
**4**	F	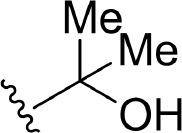	H, H	H	35.7 ± 9.5	7.447
**6**	F	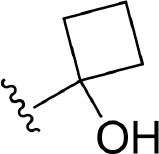	H, H	H	20.2 ± 4.7	7.695
**7**	F	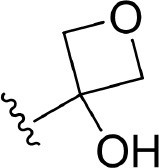	H, H	H	4,282 ± 1015	5.368
**8**	F	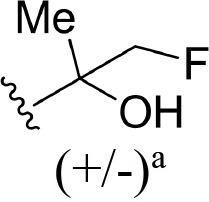	H, H	H	8.7 ± 3.5	8.060
**9**	F	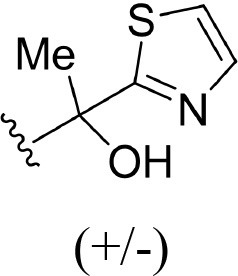	H, H	H	1.5 ± 0.7	8.824
**10**	F	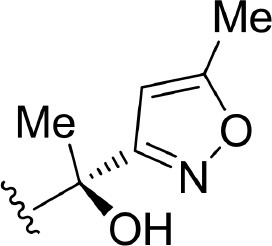	H, H	H	0.3 ± 0.3	9.523
**11**	F	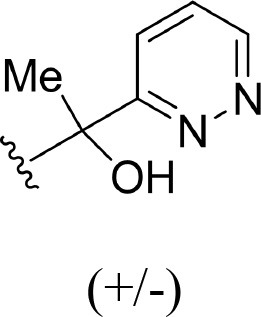	H, H	H	3.2 ± 1.7	8.495
**12**	F	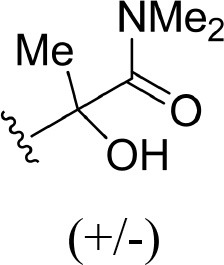	H, H	H	986 ± 375	6.006
**13**	F	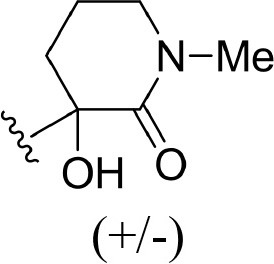	H, H	H	3 ± 1.4	8.523
**14**	F	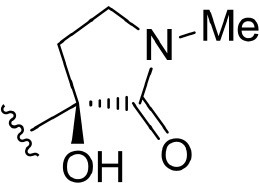	H, H	H	0.8 ± 0.8	9.097
**15**	F	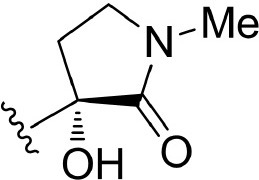	H, H	H	405 ± 272	6.393
**18**	F	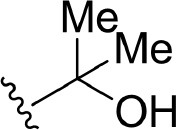	H, H	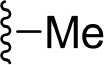	35.5 ± 19.4	7.450
**19**	F	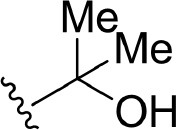	H, H	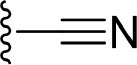	201 ± 82	6.697
**20**	F	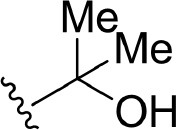	H, H	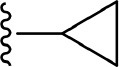	41 ± 6	7.387
**21**	F	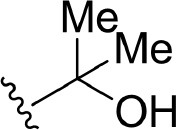	H, H	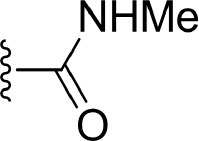	2 ± 1.4	8.699
**22**	F	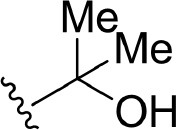	H, H	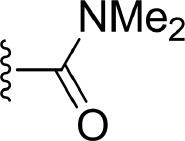	2,503 ± 726	5.602
**23**	F	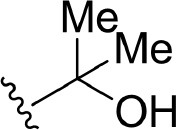	H, H	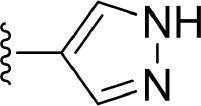	7 ± 2.8	8.155
**24**	F	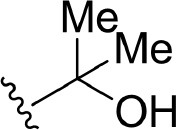	H, H	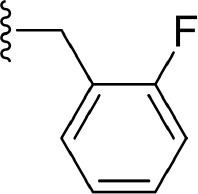	7.5 ± 6.3	8.125
**25**	F	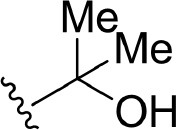	H, H	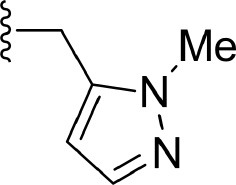	9.9 ± 2.6	8.004
**26**^b^	H	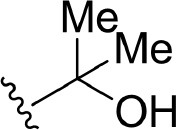	Me, H	H	547 ± 105	6.262
**27**^b^	H	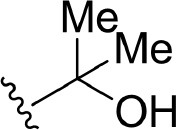	Me, H	H	1,164 ± 169	5.934
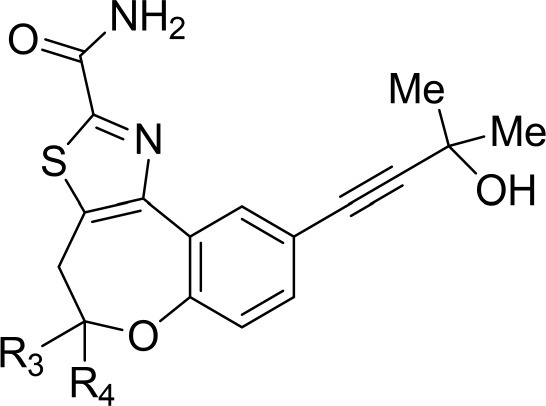						
**Compound**			**R3, R4**		**NIK ADP-FP Ki (nM)**	**pKi**
**5**			H, H		28 ± 7	7.553
**28**			H, OH (+/−)		101 ± 11	6.996
**29**			F, F		40 ± 25	7.398
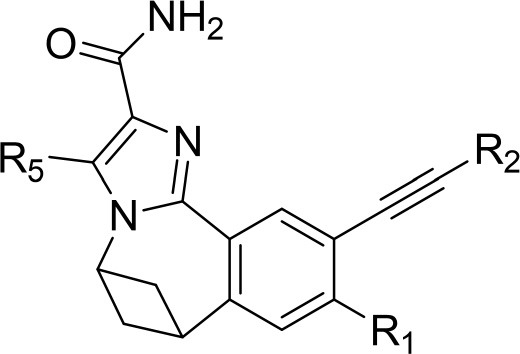						
**Compound**	**R1**	**R2**		**R5**	**NIK ADP-FP Ki (nM)**	**pKi**
**30**	H	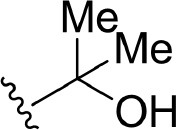		H	20 ± 9.8	7.699
**31**	F	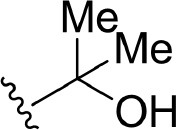		H	2.9 ± 2.2	8.538
**32**	F	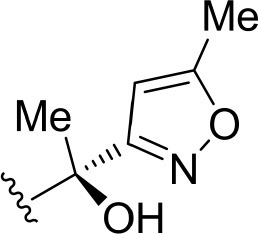		H	0.4 ± 0.4	9.398
**33**	H	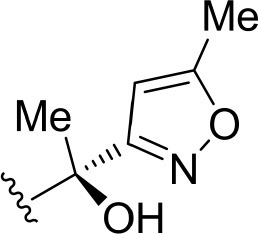		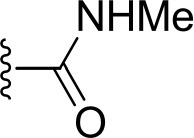	0.47 ± 0.27	9.328

a *Mixture of enantiomers*.

b *Single enantiomers of unknown absolute stereochemistry*.

### Generation of Representative Protein Conformations by MD Simulations

All three crystal structures (PDB entries: 4G3E, 5T8O, and 4IDV) were used as the initial structures to generate the representative protein conformations by MD simulations. The partial charges of the ligands were assigned with the restrained electrostatic potential (RESP) (Bayly et al., 1993) fitting algorithm based on the electrostatic potentials produced by Gaussian09 (Frisch et al., 2009) at the Hartree-Fock (HF)/6-31G^*^ level. The partial charges and the force field parameters of the ligands were yielded by the *antechamber* module implemented in AMBER16 (Case et al., 2016). The AMBER *ff03* force field (Duan et al., 2003) and the general AMBER force field (*gaff*) (Wang et al., 2004) were employed for the proteins and ligands, respectively. The *tleap* program was utilized to produce the topology and parameter files, and appropriate amounts of counter ions were added to neutralize the unbalanced charge. Each protein-ligand complex was solvated in a cuboid box of TIP3P (Lee and Duan, 2004) water molecules which were extended 12 Å from any solute atom.

Prior to the MD simulations, a three-stage minimization was carried out to remove geometric strain and close intermolecular contacts in each system. The workflow of the three-stage minimization was described as followed: (1) 1,000 cycles (500 cycles of steepest descent and 500 cycles of conjugate gradient minimization) with a 50.0 kcal/(mol·Å^2^) restraint on all heavy atoms; (2) 1,000 cycles (500 cycles of steepest descent and 500 cycles of conjugate gradient minimization) with the constrain reduced to 10.0 kcal/(mol·Å^2^); (3) 5,000 cycles (1,000 cycles of steepest descent and 4,000 cycles of conjugate gradient minimization) without any constraints. Then, each system was slowly heated from 0 to 300 K over 50 ps in the *NVT* ensemble with a 5.0 kcal/(mol·Å^2^) restraint placed on all heavy atoms, followed by 50 ps MD simulations at 300 K with the same restraint used for the equilibrium of the system. The Langevin dynamics temperature scheme was adopted to control the temperature, and the isotropic position-scaling algorithm was employed for pressure regulation. Finally, 100 ns *NPT* (*T* = 300 K and *P* = 1 atm) MD simulations were conducted. All the above MD simulations were performed by using the *pmemd* module in AMBER16. A cut-off of 8.0 Å was utilized to handle the short-range electrostatic and van der Waals interactions, while the long-range electrostatic interactions were calculated by the particle-mesh Ewald (PME) algorithm (Toukmaji et al., 2000). The SHAKE algorithm (Ryckaert et al., 1977) was applied to constrain all bonds involving the hydrogen atoms and the time step was set to 2 fs. The coordinates were saved every 10 ps, and a total of 10,000 frames were got in the end.

Eventually, all the extracted 10,000 frames of conformations were clustered by using the *cpptraj* (Roe and Cheatham, 2013) module in AMBER16 with the hierarchical agglomerative (bottom-up) algorithm. The RMSD of the pocket, which was determined by 5 Å within the ligand, was used as a clustering standard, and 10 representative conformations were finally generated from each cluster.

### Molecule Docking Calculations

Three RRD programs, including Glide (Friesner et al., 2004), Autodock Vina (Trott and Olson, 2010), and GOLD (Jones et al., 1997), were used to predict the binding mode of each ligand. Another docking method implemented in Schrödinger, named IFD (Sherman et al., 2006), was also used. All the parameters of the above docking protocols were set with no tuning of the optional parameters, unless otherwise noted as followed.

#### Glide

Two scoring modes, namely standard precision (SP) and extra precision (XP), were adopted. The binding box was defined with the centroid of the co-crystallized ligand, and its size was set to 10 × 10 × 10 Å. Then, based on the generated grid, the Glide docking calculations with the SP or XP scoring were performed.

#### Autodock Vina

Proteins and ligands were firstly converted into the *pdbqt* formats by AutoDockTools (Morris et al., 2009), along with the addition of hydrogen atoms, assignment of Gasteiger charges and cleanup of unwanted elements. The binding site was determined with the center of co-crystallized ligand, and the size of search space was set to 30 × 30 × 30 Å.

#### GOLD

Proteins were prepared by the built-in protein preparation function, and the binding site was defined by all the atoms within 6 Å around the co-crystalized ligand. The genetic algorithm (GA) method with “automatic” settings, and all the four scoring functions implemented in GOLD, including Piecewise Linear Potential (CHEMPLP), GoldScore, ChemScore, and Astex Statistical Potential (ASP), were utilized for sampling and scoring, respectively.

#### Induced-Fit Docking (IFD)

In this protocol, an initial Glide docking was firstly conducted, followed by minimization of the residues within 5 Å of any ligand pose with Prime to incorporate the conformation flexibility of the protein. Then, a Glide redocking was carried out to dock the ligand into the induced-fit receptor structure, from which the final docking score was obtained to estimate the binding energy. Both the SP and XP scoring modes were used.

#### Ensemble Docking

Based on the 30 representative conformations produced by MD simulations, all above three RRD methods were used to get an ensemble result.

### MM/PB(GB)SA Binding Free Energy Calculations and Free Energy Decomposition

The docking results with the best prediction accuracy, where all obtained ligand poses must have a similar orientation with the co-crystallized ligand, were utilized as the initial structures for this section. Then, 20 ns MD simulations for each protein-ligand complex with the procedure similar to the section named “Generation of representative protein conformations by MD simulations,” were carried out. Hundred snapshots were extracted from the final stable 4 ns MD trajectory to calculate the MM/PBSA or MM/GBSA binding free energy. The formulae were described as followed [(1)–(4)] (Wang et al., 2006; Hou and Yu, 2007; Hou et al., 2008, 2009, 2011a,b,c, 2012; Shen et al., 2013a; Xu et al., 2013; Sun et al., 2014a,b, 2018; Wang Q. et al., 2016; Zheng et al., 2017; Shi et al., 2018; Wang and Zheng, 2018; Xue et al., 2018):

(1) $$\begin{array}{l} \Delta G_{bind}=G_{com}-\left(G_{rec}+G_{lig}\right) \end{array}$$

(2) $$\begin{array}{l} \Delta G_{bind}=\Delta H-T\Delta S\approx \Delta E_{MM}+\Delta G_{sol}-T\Delta S \end{array}$$

(3) $$\begin{array}{l} \Delta E_{MM}=\Delta E_{int}+\Delta E_{ele}+\Delta E_{vdw} \end{array}$$

(4) $$\begin{array}{l} \Delta G_{sol}=\Delta G_{GB/PB}+\Delta G_{SA} \end{array}$$

(5) $$\begin{array}{l} \Delta G_{SA}=\lambda \text{SASA}+\text{b} \end{array}$$

where Δ*G*_*bind*_ refers to the total free energy upon protein-ligand binding, and it can be decomposed into three energy terms, including the gas-phase interaction energy (Δ*G*_*MM*_), the solvation energy (Δ*G*_*sol*_), and the change in the conformation entropy upon binding (–*T*Δ*S*). Δ*G*_*MM*_ is made up of intra-molecular interactions (Δ*G*_*MM*_), electrostatic interactions (Δ*G*_*ele*_) and van der Waals interactions (Δ*G*_*edw*_), whereas Δ*G*_*sol*_ contains the polar (Δ*G*_*GB*/*PB*_) and the nopolar (Δ*G*_*SA*_) contributions. In this study, the modified GB model proposed by Onufriev (igb = 2) (Onufriev et al., 2004) and the PB model using the *pbsa* program (Luo et al., 2002; Tan et al., 2006) of AMBER16, were employed to estimate the polar solvation energy. As interior dielectric constant may exert a deep influence on prediction accuracy (Hou et al., 2011b,c; Xu et al., 2013; Sun et al., 2014a,b, 2018), an interior dielectric constant of 1, 2, or 4 was tried for both the polar solvation energy (Δ*G*_*GB*_ and Δ*G*_*PB*_) calculations. The exterior dielectric constant was set to 80. The non-polar component was calculated with a linear function of solvent-accessible surface area (SASA) by using the LCPO algorithm (Weiser et al., 1999), which was shown as (5), where the λ and b were set to 0.0072, 0, and 0.00542, 0.92 in MM/GBSA and MM/PBSA calculations, respectively. Due to the expensive computational cost and low prediction accuracy under most circumstances, entropy contributions were ignored here (Sun et al., 2018).

The free energies obtained above with the best prediction accuracy (Pearson correlation coefficient) were then decomposed into individual residue-ligand contributions to further explore the interactions between NIK inhibitors and each residue. Each ligand-residue contribution can be divided into four components: van der Waals contribution (Δ*G*_*edw*_), electrostatic contribution (Δ*G*_*ele*_), polar solvation contribution (Δ*G*_*GB*/*PB*_), and non-polar solvation contribution (Δ*G*_*SA*_). All the above binding free energy calculations and free energy decomposition were performed by using the *mm_pbsa* module implemented in AMBER16.

## Results and Discussion

### Assessment of Different Docking Protocols Toward 21 Tricyclic Type I^1/2^ NIK Inhibitors

**Figure 2:**
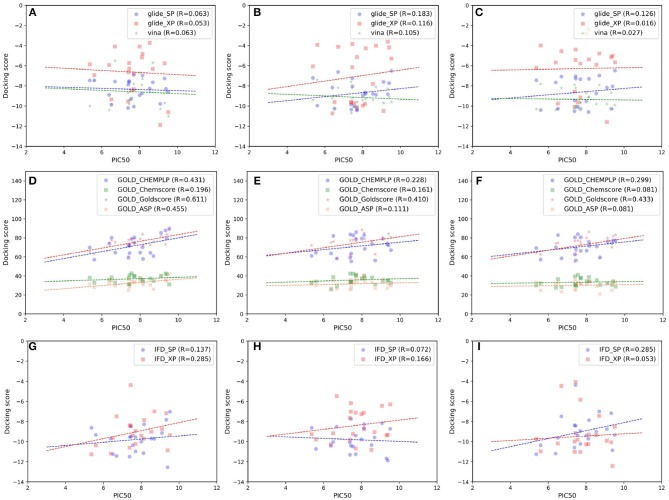
Scatter plots of experimental binding affinities (pIC_50_) vs. docking scores predicted by several docking tools based on different crystal structures, including **(A,D,G)** 4G3E, **(B,E,H)** 5T8O, **(C,F,I)** 4IDV.

**Figure 3:**
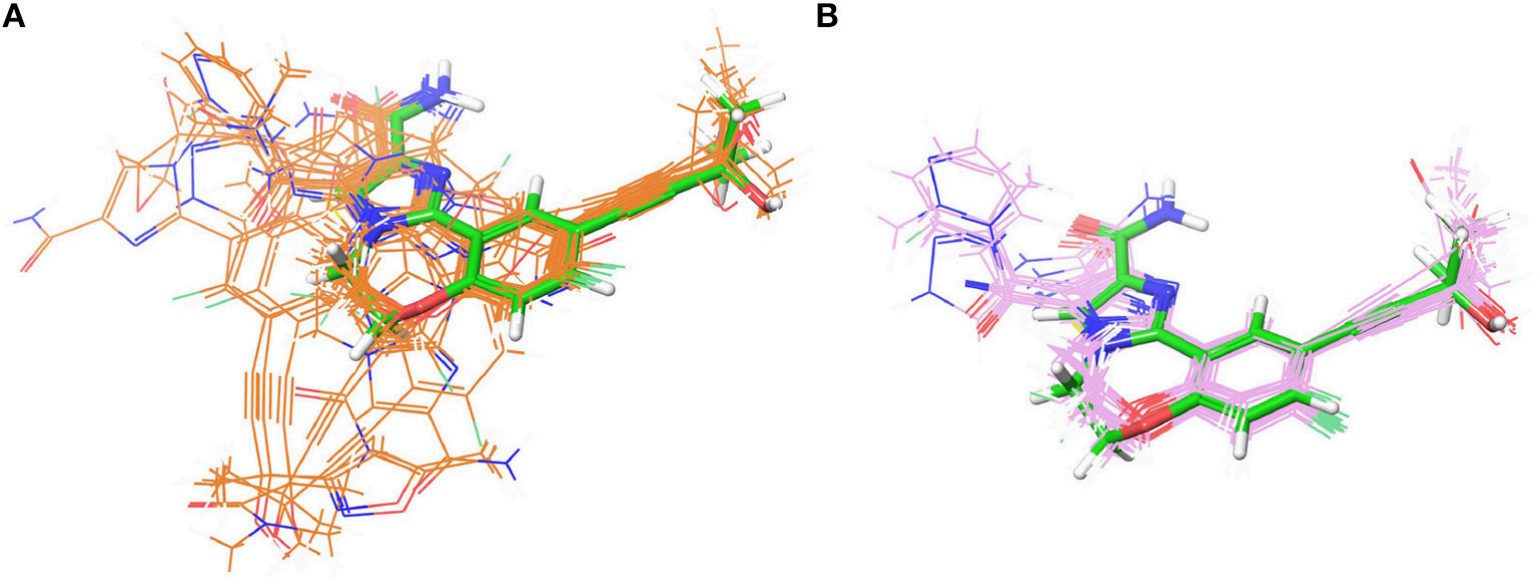
The superimposition of the docking poses of 21 type I^1/2^ NIK Inhibitors predicted by **(A)** Glide_SP and **(B)** GOLD_Goldscore based on 5T8O. The co-crystallized ligand is depicted in green.

As indicated in the previous studies, docking accuracy may be deeply rely on the docking methods or the initial conformations of crystal structures (Cross et al., 2009; Tian et al., 2013a,b, 2014; Shen et al., 2015; Wang Z. et al., 2016; Tian S. et al., 2017). So in this study, three RRD tools (Glide, Autodock Vina, and GOLD) and three crystal structures (4G3E, 5T8O, and 4IDV) were firstly assessed. The scatter plots and docking accuracy (Pearson correlation coefficient R) of the experimental binding affinities (pIC_50_) vs. docking scores predicted by different RRD tools based on different crystal structures are illustrated in Figures 2A–F. Unfortunately, except several combinations such as Goldscore based on the all three crystal structures (*R*: 0.611, 0.410, and 0.433 for 4G3E, 5T8O, and 4IDV, respectively), or CHEMPLP and ASP based on 4G3E (*R*: 0.431 and 0.445, respectively), it seems to be no significant linear correlation between the prediction and experimental activities in most cases. As far as we know, the fact that some docking methods (or scoring functions) cannot successfully sample the correct binding poses of the selected compounds may primarily account for this situation. Taking 5T8O as an example (Figure 3), when Glide_SP is adopted, the binding poses of 21 NIK inhibitors vary a lot, and some of them even locate outside of the ATP-binding pocket. However, when Goldscore is used, each pose possesses a similar orientation with the co-crystallized ligand, thus obviously enhancing the chance to get a better docking accuracy. As for the unsatisfactory sampling power, one important explanation is the steric conflicts caused by the conformational change of the surrounding residues, which in turn impedes the docking of the derivatives.

Then, the IFD implemented in Schrödinger, which considered the conformation flexibility of the residues within a given distance of the ligand, was tried. As shown in Figures 2G–I, although IFD seems to perform a little better than the corresponding RRD methods (Glide_SP or XP), its results still cannot correctly rank the experimental affinities well. As for this, the major reason may be the bad performance of Glide in our system. When conducting the IFD, an initial Glide docking is needed to roughly determine the initial location of the docked ligand. If the pose of the docked ligand cannot be sampled correctly, evidently the following “induced-fit” procedure does not make any sense. Therefore, when we carry out a molecule docking toward type I^1/2^ inhibitors or even other systems, a correct sampling orientation must be ensured firstly to get a more convincing result.

### Incorporating Receptor Flexibility by Ensemble Docking

**Figure 4:**
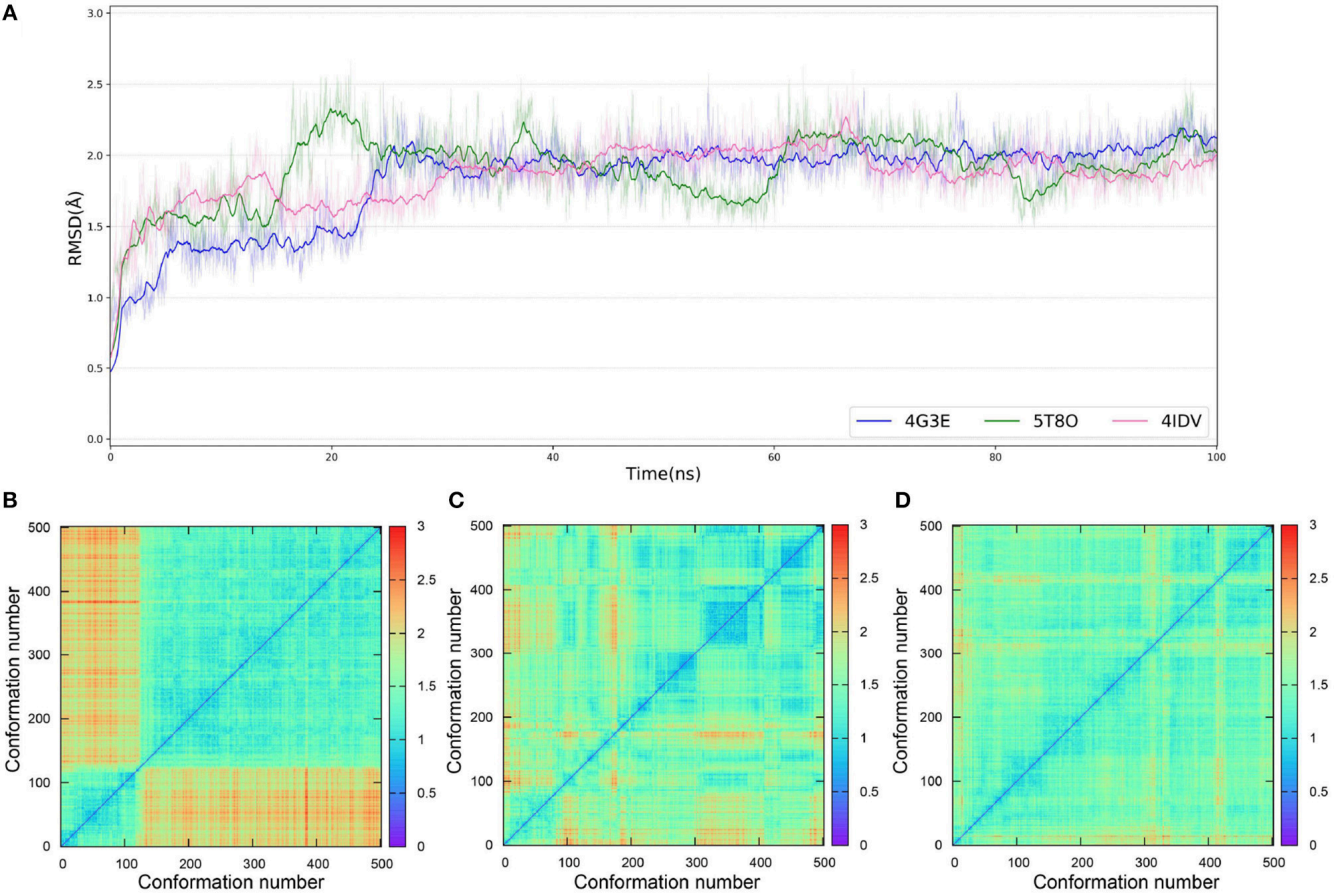
**(A)** RMSDs of the heavy atoms of the pockets defined by 5 Å within the ligand as a function of simulation time. **(B–D)** RMSD maps for the 500 representative conformations extracted from the MD trajectories based on the 4G3E, 5T8O, and 4IDV, respectively.

**Figure 5:**
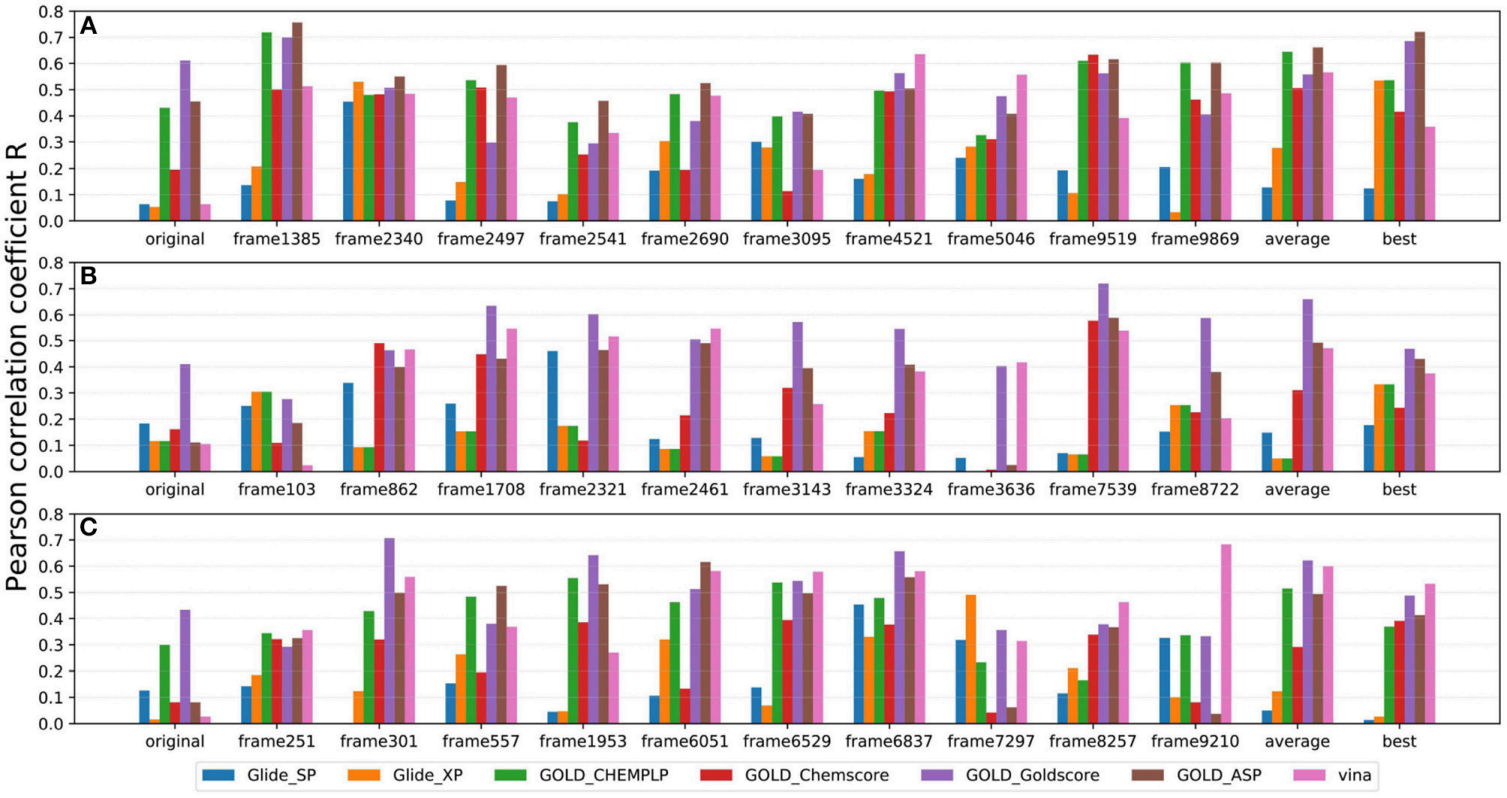
Pearson correlation coefficients of experimental binding affinities (pIC_50_) vs. docking scores predicted by several docking tools based on 10 representative protein conformations generated from **(A)** 4G3E, **(B)** 5T8O, and **(C)** 4IDV.

As IFD only allows local movements of some selected residues in the active site and its accuracy largely depends on the precision of Glide, using an ensemble of protein structures that can account for the full receptor flexibility during docking seems to be an effective approach to capture the overall influences of ligand binding on the conformations of a protein (Bowman et al., 2007; Totrov and Abagyan, 2008; Campbell et al., 2014; Ganesan et al., 2017). Thus, based on three X-ray crystal structures (4G3E, 5T8O, and 4IDV), 100 ns MD simulations were carried out and 10 representative protein conformations for each crystal structure were extracted by using the agglomerative (bottom-up) clustering algorithm. The RMSDs of the heavy atoms of the pockets defined by 5 Å within the ligand as a function of simulation time, and the RMSD maps for the 500 representative conformations extracted from the MD trajectories of these three systems are presented in Figure 4. Then, 21 type I^1/2^ NIK inhibitors were docked into all the 30 generated conformations with the method of Glide, GOLD, or Autodock Vina. The absolute values of the Pearson correlation coefficients between experimental binding affinities and docking scores are depicted as Figure 5. Overall, the docking results based on the protein conformations produced by MD simulations perform better than those obtained based on the original crystal structures. When the original crystal structure is used, the best *R* equals to 0.611 (Goldscore based on 4G3E), but when the 1,385th conformation of 4G3E is adopted, the *R*s of CHEMPLP, Goldscore, and ASP can even reach up to 0.719, 0.700, and 0.757, respectively. Then, in terms of 5T8O and 4IDV, using the conformations from the MD simulations in molecule docking is more efficient. To be specific, based on the native conformations, most docking methods show relatively poor correlations (the best are 0.410 and 0.433, respectively), but when it comes to the generated conformations, the best *R*s can be up to 0.720 (Goldscore based on the 7,539th conformation of 5T8O) and 0.707 (Goldscore based on the 301th conformation of 4IDV).

However, as for the above 30 representative conformations, not all of them display satisfactory performance (such as the 103th and the 3636th conformations of 5T8O). As for this, some bad steric conflicts between the docked ligand and the surrounding residues, which result from the stochastic MD simulations, may mainly account for it. Therefore, considering the difficulty to select the best conformation to use in the real scenario, two most common ensemble-based strategies, of which the average value and the best value of 10 docking scores were used as the final result, were tried. Just as demonstrated in Figure 5, no matter which crystal structure is chosen as the initial conformation, a remarkable improvement can be seen with the “average” or “best” strategy in most of the docking methods, where the best *R*s of the two above strategies can reach up to 0.662 (ASP based on 4G3E) and 0.721 (ASP based on 4G3E), respectively. Thus, as we can see, the ensemble docking based on MRCs generated from MD simulations surely has its own superiority over conventional molecule docking based on a single X-ray structure in our system. So when conventional virtual screening does not work well for the discovery of novel type I^1/2^ NIK inhibitors, introducing ensemble docking into virtual screening might be an appropriate option.

As far as we know, no universal docking programs (or score functions) can be adaptable for all protein-inhibitor systems (Tuccinardi et al., 2014). Indeed, in this system, Glide (whether SP or XP), which is considered as one of the most widely-used docking protocols, does not suit well for the prediction of the binding of type I^1/2^ NIK inhibitors, and the linear correlation can hardly be observed in most cases even though the ensemble docking is taken. That is to say, when we conduct a molecule docking and especially a docking-based virtual screening for type I^1/2^ NIK inhibitors, more meticulous assessment needs to be taken. Then, as for the scoring functions implemented in GOLD, Goldscore outperforms the others in terms of the overall effects, and especially when the original crystal structure is used. This may be mainly due to the fact that the effects of poor H-bond geometry and close non-bonded contacts are artificially down-weighted in Goldscore, thus weakening the influence of the steric conflicts generated from the incorrect residues and increasing the chance to get a correct docking pose. Therefore, Goldscore is probably a good choice to predict derivative binding positions when protein flexibility has a conspicuous influence on docking accuracy in some specific systems. Finally, as for Autodock Vina, one interesting thing is that compared with initial crystal structures, Pearson correlation coefficients can be improved a lot if the conformations extracted from MD simulations are utilized, which verifies the importance of the incorporation of protein flexibility in our system as well.

### MD Simulations and Binding Free Energy Calculations

**Figure 6:**
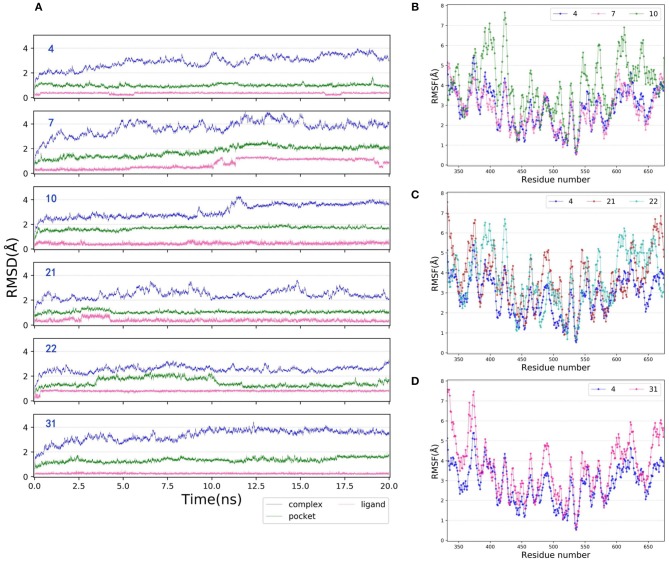
**(A)** RMSDs of the heavy atoms of the representative inhibitor-NIK complexes (**4**, **7**, **10**, **21**, **22**, and **31**), the pockets defined by 5 Å within the ligand and the ligands as a function of simulation time. **(B–D)** RMSFs of each residue of the selected complexes (**4**, **7**, **10**, **21**, **22**, and **31**) obtained from the last 4 ns MD simulations.

MD is a widely-used computational technique that can provide additional insights into time-dependent configurational changes of the structures, which is crucial for correct prediction of ligand binding and related thermodynamic and kinetic property calculations in biological systems (Mortier et al., 2015; De Vivo et al., 2016; Ganesan et al., 2017). Herein, to better explore the binding mechanism of type I^1/2^ NIK inhibitors, 20 ns MD simulations were carried out for each molecule, in which the initial conformation was determined by the docking pose based on 5T8O. The RMSDs of the heavy atoms of the six representative protein-ligand complexes (**4**, **7**, **10**, **21**, **22**, and **31**), the binding pockets defined by 5 Å within the ligand and the bound ligands among the whole 20 ns are depicted as Figure 6A. As we can see, all of them verge on stability after ~12.5 ns MD simulations, therefor the final 4 ns trajectories are chosen for further analysis so that all of the systems are guaranteed to reach the equilibrium states. The root-mean-square fluctuations (RMSFs) vs. per residue of the selected systems are illustrated in Figures 6B–D, in which all of the curves show the similar fluctuation trend, suggesting that the protein is stable enough for further exploration. As for the RMSFs, the regions with great fluctuation always refer to the residues located in the flexible loops, while the residues close to the bound ligand, which are supposed to have strong interactions with the ligand, always show low fluctuation.

**Figure 7:**
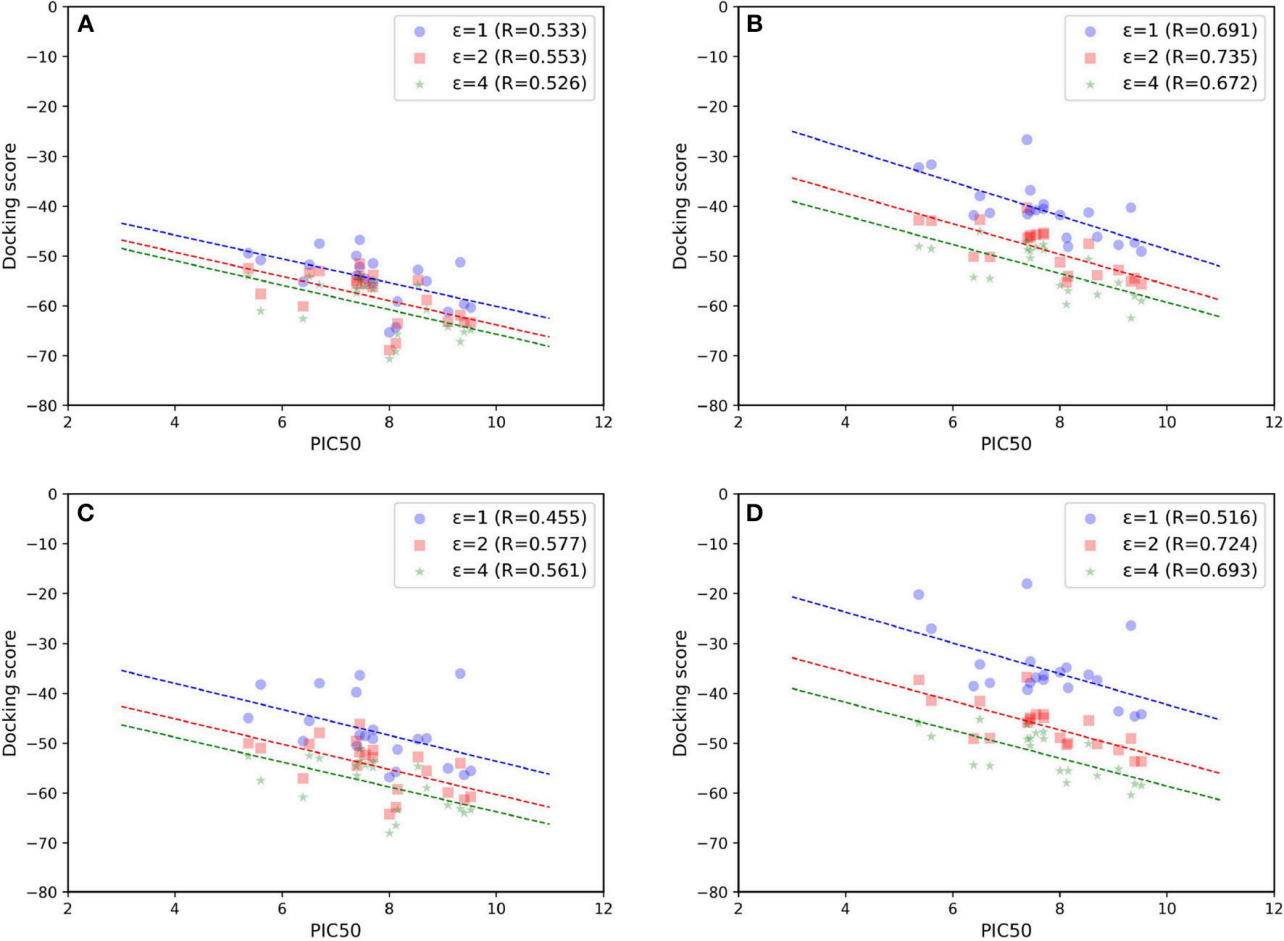
Scatter plots of experimental binding affinities (pIC_50_) vs. binding free energies predicted by **(A,B)** MM/GBSA or **(C,D)** MM/PBSA. **(A)** and **(C)** are based on minimized structures while **(B)** and **(D)** are based on the final stable 4 ns MD trajectories.

As most scoring functions used in molecule docking do not have much physical significance, more valid free energy calculation approaches that can better account for the binding affinity are also needed in this study. Although the absolute total energies predicted by MM/GB(PB)SA cannot reproduce the actual experimental values at all due to the calculation error of the entropy contribution, they can still be shown to well reflect the relative difference in some targets (Tian Y. et al., 2017; Tang et al., 2018). Besides, compared with the theoretically rigorous methods such as thermodynamic integration (TI) and free energy perturbation (FEP), MM/GB(PB)SA is much more time-saving and comparatively effective, thereby leading to their frequent use in a number of previous studies (Pan et al., 2013; Shen et al., 2013b; Sun et al., 2013; Wang et al., 2014; Kong et al., 2015, 2016, 2017, 2018; Xu et al., 2016; Tian Y. et al., 2017). Hence, based on above 20 ns MD simulations, MM/GBSA and MM/PBSA were performed in our system. The scatter plots of experimental binding affinities vs. binding free energies are shown in Figures 7B,D, respectively. As comparison, the free energies only based on the minimized structures are also calculated and exhibited in Figures 7A,C. On the whole, MM/GBSA and MM/PBSA work considerably well in this system, and MM/GBSA performs relatively a little better. Then, compared with the corresponding docking result (Goldscore based on 5T8O), MM/GB(PB)SA rescoring can surely improve the prediction accuracy, suggesting that it is most likely suitable to use it as a rescoring tool to further guide the discovery of novel molecules. The results calculated after MD simulations appear to be significantly better than the ones obtained from minimized structures, which highlights the enormous impact of protein flexibility on ligand binding toward type I^1/2^ NIK inhibitors as well. Finally, just as previous evidence shows, dielectric constant indeed have a great influence on the prediction accuracy in this system, and the results are the best in our hands when interior dielectric constant is set to two, with best Rs reaching up to 0.735 and 0.724 by using the method of MM/GBSA and MM/PBSA, respectively.

Another advantage of MM/PB(GB)SA is that they allow for the decomposition into identifiable interaction terms, which can be further explored separately to get insights into driving forces for novel and potent binders. The binding free energies predicted by MM/GBSA (ε = 2) and the individual energy terms are enumerated in Table 2. As we can see, van der Waals interactions (Δ*G*_*vdw*_) dominate the binding of the inhibitors, but the total polar contributions, which are composed of electrostatic interactions (Δ*G*_*ele*_) and the polar contribution of solvation effect (Δ*G*_*GB*_), are perhaps also responsible for the discrepancy of the binding affinities of this series of analogs. Then, one interesting thing is that the total polar contributions are found to be unfavorable for the binding with all the values displaying positive. As for this, the fact that the strong H-bonds interactions between the ligands and the receptors still cannot compensate the large desolvation penalties during the binding may mainly account for it. When it comes to the total binding free energies (Δ*G*_*total*_), most of the alkyne substitutions (**4**, **6**, **7**, **10**, **14**, and **15**), imidazole 5-substitutions (**18**–**25**) and [4.1.1] bicyclic replacements (**30**–**33**) indeed have a great effect on the predicted activities, which is roughly consistent with the actual experimental results.

**Table 2:** Binding free energies predicted by MM/GBSA (ε = 2).

**Compound**	**ΔG**_ele_^a^	**ΔG**_vdw_^b^	**ΔG**_SA_^c^	**ΔG**_GB_^d^	**ΔG**_bind_^e^	**pIC**_**50**_
**3**	−15.42 ± 0.26	−42.86 ± 0.31	−4.07 ± 0.02	19.64 ± 0.23	−42.71 ± 0.26	6.509
**4**	−13.18 ± 0.29	−46.94 ± 0.28	−4.24 ± 0.01	18.08 ± 0.18	−46.27 ± 0.24	7.447
**5**	−17.49 ± 0.30	−45.83 ± 0.32	−4.24 ± 0.01	21.82 ± 0.22	−45.73 ± 0.27	7.553
**6**	−12.93 ± 0.26	−45.48 ± 0.26	−4.21 ± 0.01	17.27 ± 0.18	−45.36 ± 0.22	7.695
**7**	−12.45 ± 0.26	−48.35 ± 0.28	−4.48 ± 0.02	22.45 ± 0.23	−42.83 ± 0.29	5.368
**10**	−16.74 ± 0.25	−56.63 ± 0.30	−5.06 ± 0.01	22.75 ± 0.15	−55.67 ± 0.26	9.523
**14**	−19.36 ± 0.33	−52.69 ± 0.34	−4.62 ± 0.01	23.82 ± 0.22	−52.84 ± 0.29	9.097
**15**	−14.20 ± 0.41	−53.17 ± 0.35	−4.72 ± 0.02	21.95 ± 0.27	−50.14 ± 0.33	6.393
**18**	−17.93 ± 0.39	−49.79 ± 0.28	−4.47 ± 0.01	26.30 ± 0.30	−45.89 ± 0.26	7.450
**19**	−20.13 ± 0.25	−53.49 ± 0.24	−4.72 ± 0.01	28.18 ± 0.18	−50.16 ± 0.23	6.697
**20**	−8.72 ± 0.27	−49.09 ± 0.26	−4.28 ± 0.02	21.78 ± 0.29	−40.31 ± 0.25	7.387
**21**	−18.81 ± 0.40	−55.95 ± 0.31	−5.01 ± 0.01	25.87 ± 0.24	−53.90 ± 0.24	8.699
**22**	−9.61 ± 0.24	−49.16 ± 0.34	−4.52 ± 0.02	20.35 ± 0.20	−42.94 ± 0.30	5.602
**23**	−22.27 ± 0.33	−54.19 ± 0.35	−5.06 ± 0.02	27.49 ± 0.25	−54.04 ± 0.29	8.155
**24**	−10.51 ± 0.29	−58.38 ± 0.29	−5.30 ± 0.01	18.92 ± 0.20	−55.27 ± 0.29	8.125
**25**	−12.18 ± 0.24	−55.17 ± 0.30	−4.92 ± 0.02	21.06 ± 0.21	−51.21 ± 0.26	8.004
**29**	−19.91 ± 0.25	−46.18 ± 0.34	−4.27 ± 0.02	24.03 ± 0.16	−46.34 ± 0.30	7.398
**30**	−15.77 ± 0.22	−47.00 ± 0.27	−4.16 ± 0.01	21.24 ± 0.17	−45.68 ± 0.25	7.699
**31**	−13.13 ± 0.30	−48.94 ± 0.27	−4.36 ± 0.01	18.90 ± 0.19	−47.53 ± 0.21	8.538
**32**	−16.59 ± 0.31	−56.01 ± 0.34	−4.99 ± 0.01	23.12 ± 0.19	−54.47 ± 0.29	9.398
**33**	−12.97 ± 0.25	−63.69 ± 0.24	−5.44 ± 0.01	27.04 ± 0.19	−55.07 ± 0.23	9.328

a *Electrostatic interaction*.

b *Van der Waals interaction*.

c *Non-polar contribution of the solvation effect*.

d *Polar contribution of the solvation effect*.

e *Binding free energy*.

*All above values are shown as mean ± standard error of mean*.

### Further Analysis of the Interactions Between NIK and Inhibitors

**Figure 8:**
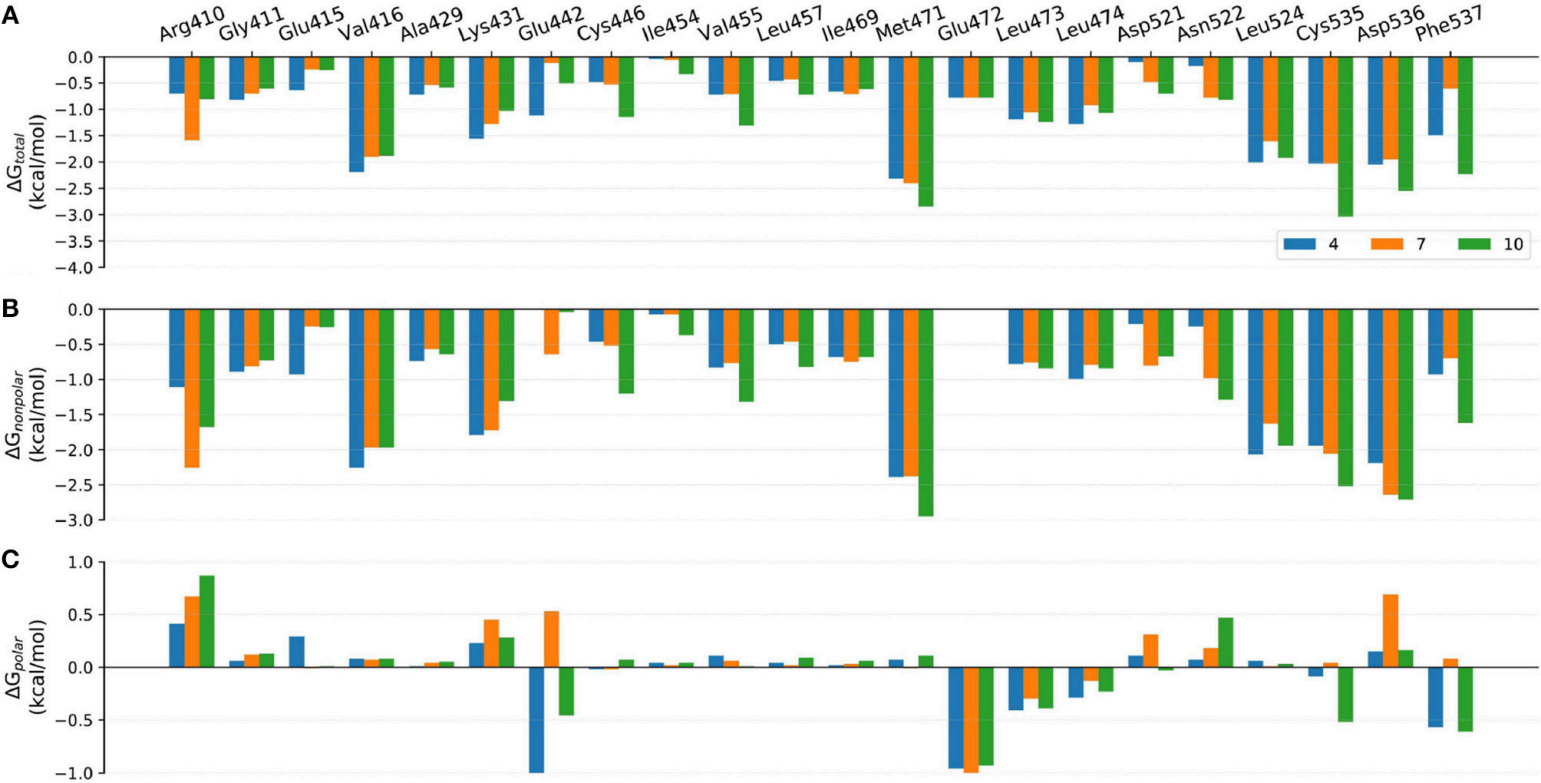
Comparison of the **(A)** binding free energies, **(B)** nonpolar contributions, and **(C)** polar contributions of some key residues of compounds **4**, **7**, and **10**.

As shown in above sections, protein flexibility may produce a significant effect on the binding of NIK and inhibitors, and conventional static structural analysis may not fully reflect the real binding characteristics, so a deeper analysis based on MD simulations toward six representative inhibitor-NIK complexes (**4**, **7**, **10**, **21**, **22**, and **31**) are conducted in this section. Firstly, the hydrogen bonds from the final stable 4 ns MD trajectories of each system were searched for by using the *cpptraj* module implemented in AMBER17 with the distance and angle cutoff set to 3.0 Å and 135°, respectively. Just as listed in Table 3, overall, the Leu474^m^ and Glu472^m^ in the hinge region, the Glu442^m^ located in the αC-helix, and the Phe537^m^ belonging to DFG motif have the largest H-bond occupancy, suggesting that these residues may be vitally important in the binding of this series of analogs. However, one interesting thing is that a series of analogs with alkyne substitutions (**4**, **7**, and **10**), have a significant discrepancy in activities but much similarities in H-bond distribution. To account for it, our preliminary speculation is that H-bond interactions may not play a decisive role in structure-activity relationship (SAR). In terms of individual compound, as we can see, structural modifications in different positions may produce a large distinction in H-bond distribution. For example, the molecules with no imidazole 5-substitutions (**4**, **7**, **10**, and **31**) tend to form H-bonds with both the Glu472^m^ and Leu474^m^ in the hinge region, whereas the others (**21** and **22**) are more likely to form H-bonds only with the Leu474^m^. As for this, the major explanation is the fact that imidazole 5-substitutions can cause the slight conformation change of the optimal binding mode, thus making the pose away from the Leu472^m^ (Figure 8B). Besides above ubiquitous H-bonds, the 5-substitutions of the imidazole ring can also yield some potential strong H-bond interactions, such as Gln481^m^ (**21)** and Ser478^m^ (**22**), which may provide some novel ideas for further structural optimization. In addition, one intriguing finding occurred with imidazole 5-substitution is that intramolecular H-bonds can be observed between two amide groups both in **21** and **22**, and their occupancies can be up to 17.05 and 97.70%, respectively. Although the formation of the intramolecular H-bond is beneficial to the maintenance of amide conformation in some ways, residue-ligand interactions can be accordingly weakened owing to the occupancy of H-bond donor or acceptor in the molecule. Therefore, considering the longstanding occupancy of intramolecular H-bond in **22**, it is easy to explain for the poorer experimental activity of **22**.

**Table 3:** Hydrogen bonds analysis of the six representative systems based on final 4 ns of the MD trajectories.

**Donor**	**Acceptor**	**Occupancy (%)^a^**					
		**4**	**7**	**10**	**21**	**22**	**31**
Leu474-NH	Ligand-O	57.80	57.80	22.00	22.95	0.95	34.20
Ligand-NH	Leu474-O	/^c^	/	/	77.90	39.55	/
Ligand-NH	Glu472-O	91.50	91.35	90.25	/	/	89.85
Ligand-OH	Glu442-OE	98.95	99.50	98.60	97.80	97.95	91.90
Phe537-NH	Ligand-O	22.30	32.40	39.05	8.55	8.50	37.75
Lys431-NH	Ligand-F	2.35	0.60	0.60	1.90	2.15	1.40
Asp536-NH	Ligand-O(N)^b^	0.10	0.05	12.95	0.45	0.25	/
Gln481-NE-H	Ligand-O	/	/	/	71.8	/	/
Ser478-NH	Ligand-O	/	/	/	0.90	21.45	/
Ligand-NH	Ligand-N	/	/	/	17.05	97.70	/

a *Only occupancy larger than 5% in at least one systems is shown*.

b *Different ligands have different groups at the same position*.

c *This H-bond cannot be observed over the all trajectories*.

**Figure 9:**
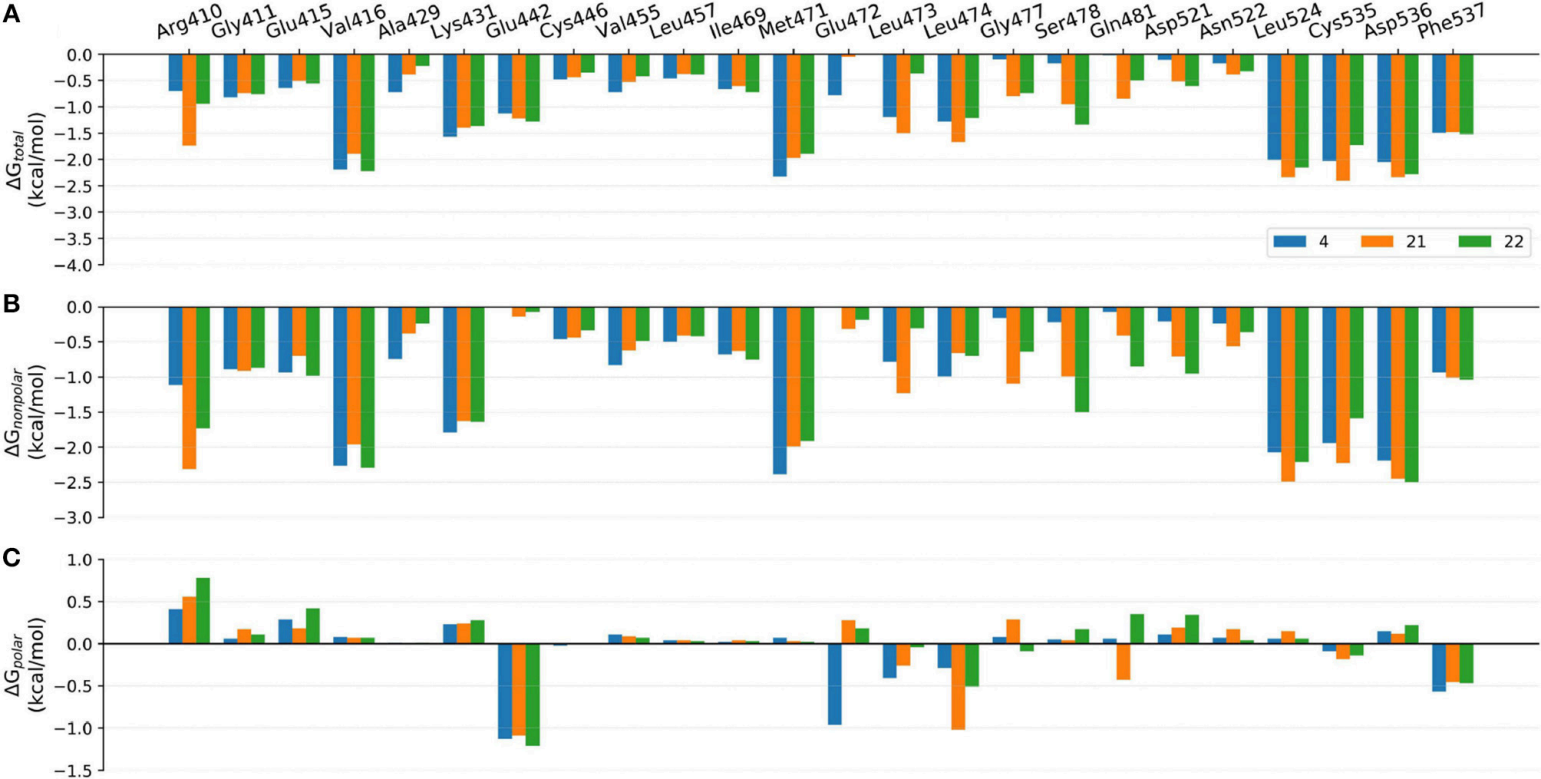
Comparison of the **(A)** binding free energies, **(B)** non-polar contributions, and **(C)** polar contributions of some key residues of compounds **4**, 1**7**, and **18**.

**Figure 10:**
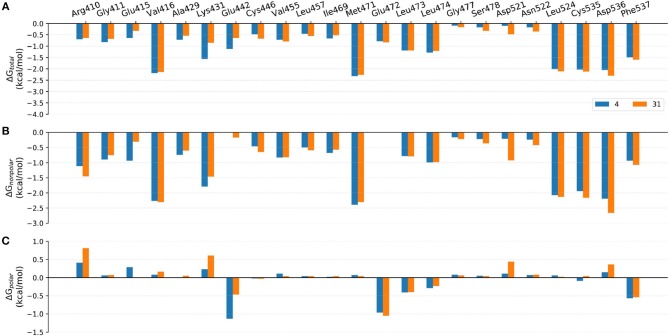
Comparison of the **(A)** binding free energies, **(B)** non-polar contributions, and **(C)** polar contributions of some key residues of compounds **4** and **31**.

Although the prediction accuracy of above mentioned MM/GB(PB)SA cannot completely satisfy our expectation (in general *R* > 0.8 is considered as a good linear correlation), it can still distinguish tight-binding and loose-binding inhibitors to some extent. Thus, to better elaborate the key residues of the binding of type I^1/2^ NIK inhibitors, the binding free energies of above six representative molecules (**4**, **7**, **10**, **21**, **22**, and **31**) predicted by MM/GBSA (ε = 2) were decomposed into the contribution of per residue. Just as shown in Figures 8–10, on the whole, Arg410^m^, Val416^m^, Lys431^m^, Gln442^m^, Met471^m^, Leu473^m^, Leu474^m^, Leu524^m^, Cys535^m^, Arg536^m^, and Phe537^m^ may contribute the most to the total binding free energies with most of the values less than −1.0 kcal/mol. As far as we know, the understanding of the distribution and types of key residues among the binding pocket could provide valuable information for the structure-based drug design (SBDD), so the properties of above key residues in terms of the overall effect are explored first. Respecting the locations of above residues, Arg410^m^, Val416^m^, Leu473^m^, Leu474^m^, Leu524^m^, and Cys535^m^ make up the ATP-binding pocket, whereas Lys431^m^, Gln442^m^, Met471^m^, Asp536^m^, and Phe537^m^ embrace around the back cavity, which means that both the ATP-binding and the back pocket play an essential role in the binding of these analogs. Then, as we have seen, different residues may produce different contributions to the binding modes. Hydrophobic residues (such as Val416^m^, Met471^m^, and Leu524^m^), or some hydrophilic residues with their hydrophobic regions located toward the ligand (such as Lys431^m^, Cys535^m^, and Asp536^m^), account for the largest proportion of the total energies, suggesting that non-polar interactions may be vitally crucial. Nevertheless, as for some residues such as Glu442^m^ and Leu472^m^, the polar interactions dominate their total contributions, implying that polar contributions such as H-bond interactions can also make some sense, which is roughly consistent with above H-bond analysis. In addition, due to the stereo conflicts and the large desolvation effects, some hydrophilic residues such as Arg410^m^, Lys431^m^, and Asp536^m^, are inclined to yield unfavorable polar contributions, which can in turn impede the binding of the ligand in a way. Therefore, to design novel type I^1/2^ NIK inhibitors with better binding affinities, these advantageous residues should be paid more attention to, and the residues that can cause adverse effects, need to be avoided as far as possible. Next, in order to probe the influence of different substitutions, a deeper comparison is made among the selected systems, and the details are listed as follow.

#### Comparison of Compounds 4, 7, and 10

**Figure 11:**
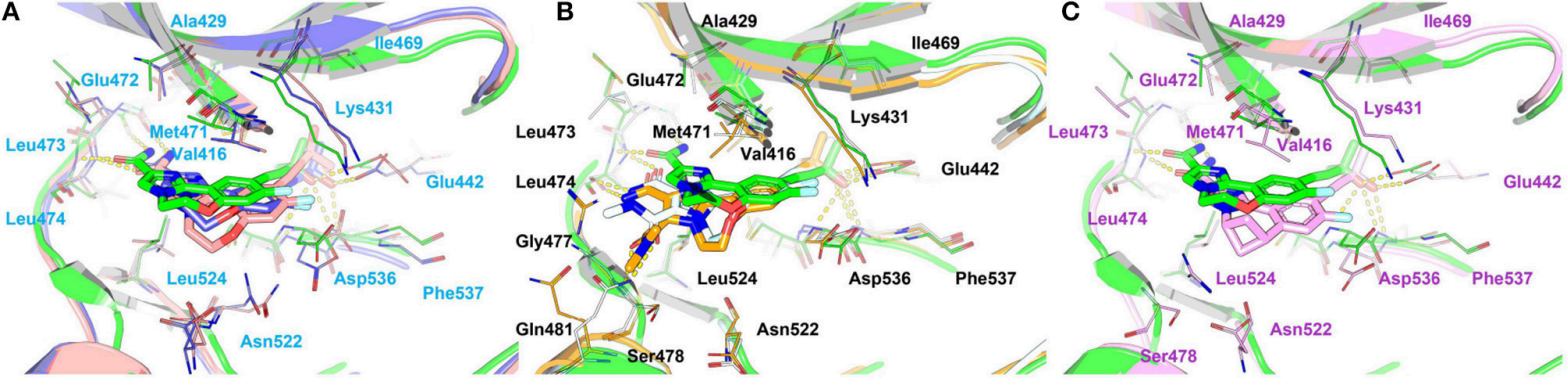
Comparison of the averaged structures of **(A)** compounds **4**, **7**, and **10**, **(B)** compounds **4**, **17**, and **18**, and **(C)** compounds **4** and **31**. **4**, **7**, **10**, **17**, **18**, and **31** are colored in green, salmon, lightblue, palecyan, orange, and pink, respectively.

The only discrepancy in compounds **4**, **7**, and **10** is the alkyne substitution, in which a propargyl alcohol, a 3-alkynyl-3-hydroxyoxetane and a (R)-3-alkynyl-3-hydroxy-5-methylisoxazole exist in **4**, **7**, and **10**, respectively. Each of them has a hydroxyl group that can form H-bonds with both the αC-helix (Glu442^m^) and the DFG motif (Phe537^m^) (Figure 11A), implying that this hydroxyl group may play a crucial role in the stabilization of these analogs in the back pocket. The binding affinities of these three molecules are predicted to −46.27, −42.83, and −55.67 kcal/mol, respectively, which have a similar trend with their experimental values. As illustrated in Figure 8, although compound **7** has no weaker non-polar interactions with the residues around the back pocket (Lys431^m^, Gln442^m^, Met471^m^, Asp536^m^, and Phe537^m^) than **4** and **10**, its polar contributions caused by the back cavity decrease a lot, especially those of the Gln442^m^ (0.53 kcal/mol vs. −1.13 and −0.46 kcal/mol), Asp536^m^ (0.69 kcal/mol vs. 0.15 and 0.16 kcal/mol) and Phe537^m^ (0.08 kcal/mol vs. −0.57 and −0.61 kcal/mol). Combined with above H-bond analysis, we speculate that compound **7** must undergo a hard process to overcome large solvation energy upon binding. Then, comparing the structures of **4** and **7**, we find that the oxetane of **7** appears more hydrophilic than the isopropyl of **4** due to the existing of an oxygen atom, which can account for the more notable desolvation effects of **7** to some extent. As for the best affinity of **10**, the enhancement of the non-polar contributions caused by the back pocket may play the most important role. On account of the addition of 5-methylisoxazole ring, not only above key residues around the back pocket, but also some other adjacent residues (such as Cys446^m^ and Val455^m^), show an increasing trend in the non-polar contributions, thereby facilitating the high activity of **10**. Besides, protein flexibility and conformational change can never be ignored in biological systems. Indeed, the contributions of some residues far away from the back pocket (such as Arg410^m^, Asp521^m^, and Asn522^m^) can also be found to contribute a bit to the activity difference of these molecules.

#### Comparison of Compounds 4, 21, and 22

The comparison of compounds **4**, **21** and **22** mainly focuses on the influence of ATP-binding pocket, and the structural modification can only be observed in imidazole 5-substitution, where the hydrogen atom of **4** is replaced by an N-methylformamide and an N, N-dimethylformamide in **21** and **22**, respectively. Because of the addition of a more hydrophobic group at imidazole 5-substitution, **21** and **22** show stronger non-polar interactions with the corresponding residues in the ATP-binding pocket, including Gly477^m^, Ser478^m^, Gln481^m^, Asp521^m^, and Asn522^m^. As for the polar contributions, although compound **4** can get strong polar contributions from Glu472^m^, **21** can interact with Leu474^m^ and Gln481^m^ more frequently, which is roughly in accord with above H-bond analysis. Based on the above reasons, it is not too surprising to see compound **21** displays a slightly better activity than **4**. When it comes to **22**, its intramolecular H-bond should be responsible for its relatively poorer affinity. On one hand, the formation of the intramolecular H-bond directly reduces the polar contributions from the hinge region (such as Glu472^m^ and Leu474^m^). On the other hand, the tendency to form an intramolecular H-bond also leads to the conformational flip of the amide group (see Figure 11B), which loses some important interactions with Gln481^m^ and Leu473^m^ as well.

#### Comparison of Compounds 4 and 31

The most innovative modification of this series of analogs must be the benzoxepin core being replaced with a bridged-bicyclic core, which can be considered as the first application of isosteric replacement of a seven-member ring with a bridged bicyclic 4.1.1 system in the context of drug design. With this creation, ~10 fold increase can be observed in experimental NIK inhibition (K_i_). As can be seen in Figure 11C, compound **31** can go through a distinct conformational shift compared with compound **4**, thus increasing the interactions with the ATP-binding pocket, especially Ser478^m^, Asp521^m^, and Asn522^m^. Of course, the interactions with the residues in the back pocket (such as Lys431^m^ and Glu442^m^) accordingly decreases, but they can still not compensate the increment of those around the ATP-binding pocket, which may mainly account for the activity difference between **4** and **31**.

### Suggestions for the Design of Novel Type I^1/2^ NIK Inhibitors

Based on all above computational methods, we would like to provide some suggestions for the design of novel type I^1/2^ NIK inhibitors. The details are summarized below.

1. The selection of docking protocols and crystal structures has a great impact on the docking accuracy toward type I^1/2^ NIK inhibitors, thereby affecting the prediction of their binding modes and relative binding affinities even if the docked molecule is an analog of the co-crystallized ligand. Therefore, it is better to conduct a systematical assessment before the SBDD, no matter whether a docking-based virtual screening or just a docking-based structural optimization.
2. Protein flexibility can never be ignored in biological systems and especially those involving allosteric effects (such as the binding of type I^1/2^ kinase inhibitors). Then, using the method of ensemble docking based on the structures extracted from MD simulations, may account for the full receptor flexibility during docking and improve the docking accuracy to some extent. Hence, virtual screening based on ensemble docking can be used as a beneficial attempt to discover novel inhibitors. Also, the static structural analysis should be replaced with the integration of MRCs, to better illustrate the binding characteristics.
3. MM/GBSA and MM/PBSA that consider both the prediction accuracy and computational efficiency simultaneously surely have their own superiority over other methods to predict the ranking of a series of molecules. So it should be a good idea to using them as a rescoring tool for virtual screening, or a relatively more reliable approach for the guidance of lead compound optimization.
4. As for the binding of type I^1/2^ NIK inhibitors, some distinct characteristics can be universally observed in most of them. Firstly, the ligands tend to form H-bonds with some specific regions, including the hinge region [Leu474^m^ and (or) Glu472^m^], the αC-helix (Glu442^m^), and the DFG motif (Phe537^m^ or Asp536^m^). So some H-bond acceptors or donors should be designed to occur at the appropriate positions, and intramolecular H-bonds that may in turn hinder the interactions with surrounding residues should be avoided if possible. Then, hydrophobic interactions showed most contribute to the binding of type I^1/2^ NIK inhibitors in our study, especially the contributions from the residues such as Val416^m^, Lys431^m^, Met471^m^, Leu524^m^, Cys535^m^, and Asp536^m^. Thus, increasing the hydrophobic interactions of these key residues is probably helpful for the increase of the binding affinities. Next, the unfavorable contributions caused by the desolvation effects (especially those in the back pocket) should also not be ignored. Accordingly, a functional group with suitable hydrophobicity should be designed to extend into the back cavity of NIK, so that the desolvation effects and the H-bond interactions can be well-balanced.

## Conclusion

An integrating computational case study, which involves molecule docking, MD simulations, ensemble docking, MM/GB(PB)SA binding free energy calculations and decompositions, has been conducted toward type I^1/2^ NIK inhibitors to further illuminate their binding mechanisms. As conventional RRD docking methods and IFD cannot well reflect the relative activity, ensemble docking by using MRCs is adopted and the docking result is largely improved, suggesting that protein flexibility is vitally important in type I^1/2^ inhibitors binding. Then, MD simulations combined with MM/GB(PB)SA binding free energy calculations are used to further explore NIK-ligand interactions. It is found that a remarkably improved linear correlation can be observed between prediction and experimental activities, implying that MM/GB(PB)SA is adaptable for the prediction of the binding of NIK and its inhibitors. Further analysis shows that hydrophobic interactions play the most essential role in ligand binding, whereas the polar contributions consisting of the electrostatic interactions and the polar contribution of solvation effect, also take some effect. Next, H-bond analysis as well as MM/GBSA decomposition is carried out and several key residues are specially revealed. A deeper comparison of several pairs of representative inhibitor derivatives illustrates that the activity difference of a series of analogs also greatly depends on the non-polar contributions. Likewise, the H-bond interactions with some key residues and the large desolvation effect in the back pocket contribute a lot to the overall affinities as well. These findings are expected to provide valuable insights into the discovery of novel type I^1/2^ NIK inhibitors.

## Author Contributions

DL and XY conceived the idea and supervised the study. CS conducted the theoretical study. CS, HL, XW, TL, EW, LX, and HY analyzed the data. CS drafted the manuscript. DL and XY edited the manuscript. All authors read and approved the final manuscript.

### Conflict of Interest Statement

Author HY was employed by company Rongene Pharma Co., Ltd. All other authors declare no competing interests.
